# Using Informatics to Build a Digital Health Footprint of Patients Living With Inherited Metabolic Disorders Identified by Newborn Screening

**DOI:** 10.1097/PHH.0000000000001250

**Published:** 2020-11-16

**Authors:** Rani H. Singh, Sheereen J. Brown, Piper M. Hale, Kristen Narlow, Saran Gurung, Mary L. Salvatore, Jimica K. Tchamako

**Affiliations:** Medical Nutrition Therapy for Prevention (MNT4P), Emory University School of Medicine, Atlanta, Georgia (Drs Singh and Gurung and Mss Narlow and Salvatore); and the Public Health Informatics Institute at the Task Force for Global Health, Decatur, Georgia (Mss Brown, Hale, and Tchamako).

**Keywords:** informatics, inherited metabolic disorders, medical nutrition therapy

## Abstract

Newborn screening is a process identifying people with inherited metabolic disorders (IMDs) at birth, but these patients are often lost to follow-up, and limited data on their long-term needs are available to advocate for policies that will help this vulnerable community. Using informatics best practices, the Medical Nutrition Therapy for Prevention (MNT4P) program and the Public Health Informatics Institute successfully deployed a minimally viable product—that is, the most basic working version that is scalable—allowing for lifelong patient follow-up and outcome and needs tracking, and that can address national data gaps. The new system offers a HIPAA-compliant, efficient record-keeping system that allows data standardization and harmonization. MNT4P staff have transitioned completely away from former manual processes and are relying on this system to log and track patient information. Other programs serving patient populations burdened with rare, marginalized diseases also may benefit from this work.

## Abstract 2

Supplemental Digital Content is Available in the Text.

In 2009, research evaluating the public health impact of newborn screening (NBS) in Georgia found that while NBS was effective at early identification of children born with inherited metabolic disorders (IMDs) and generating data on IMD diagnoses, data to support lifelong management, personalized nutrition treatment programs, and positive long-term health outcomes were lacking.^1^ Identified treatment gaps for IMD patients included access to medical nutrition therapy and medical foods.^2^

To bridge these identified gaps, the 2009 research team recommended building partnerships to support follow-up and care coordination for IMD patients identified through NBS to ensure a sustainable “diet for life.” This work highlighted the need for a program to inform long-term patient follow-up, case management, and service provision to support those living with IMDs in the state of Georgia. Ideally, this interventional program would also help supply historically untracked but much needed data around the needs of this population, including utilization rates of medical nutrition therapy.

In 2016, the Medical Nutrition Therapy for Prevention (MNT4P) program was established by Emory University School of Medicine's Department of Human Genetics, primarily using funding from the Georgia Department of Public Health.

## Program Context

MNT4P is a comprehensive nutrition program designed to serve Georgia residents living with IMDs who face financial and other barriers to obtaining necessary medical foods and related supplies and to accessing services by a trained nutrition workforce that they require to survive and thrive. IMDs are considered rare diseases; at a prevalence of about 1 per 2500 live births, an estimated 50 children are born each year in the state of Georgia with IMDs.^3^,^4^ Accessibility is a common dilemma in the IMD community, often associated with inconsistent insurance reimbursement policies both for medical foods and for nutrition services, even for those with full insurance coverage.^5^,^6^ In addition, federal and state funding can be difficult to divert to supporting accessibility, due to a lack of data on the IMD community's needs and typical use of medical supplies.

As a point of entry, MNT4P acts as a short-term bridge resource to connect patients with medical foods and monitoring supplies. MNT4P offers additional services, including assisting with insurance navigation following a payer of last resort model; providing nutrition treatment, counseling, and monitoring; and conducting quality improvement activities and knowledge generation that can inform future research and data collection under the direction of trained registered genetic metabolic dietitians.

## Building the Digital Health Footprint

MNT4P identified the need for a public health informatics infrastructure that would replace the current manual data management processes and enable long-term follow-up and knowledge generation. The Public Health Informatics Institute (PHII) defines public health informatics as, “the process by which raw data turns into information and, subsequently, knowledge.”^7^

Currently, when a newborn in the state of Georgia is identified through NBS as having an IMD, the Georgia Department of Health laboratory alerts Emory Genetics Clinic, the only clinic in Georgia that provides specialist care to patients with IMDs. If nutritional management or a related service is unmet through the clinic, the Emory Genetics Clinic will refer a patient to MNT4P. In addition, some patients identified symptomatically by other clinical providers in Georgia prior to the return of NBS test can refer patients to MNT4P for support. This process marks the beginning of the patient record with MNT4P and establishes a relationship between the MNT4P program and a patient living with an IMD. Patients who are not diagnosed at birth or who move into the state from elsewhere may be referred at a later age to MNT4P directly by their health care provider or through the Emory Genetics Clinic. Establishing a permanent, evolving “digital health footprint” ensures that patients with IMDs have the opportunity for lifelong access to medical foods and nutrition resources, along with continual monitoring, and enables MNT4P to manage patient health on an individual and aggregate level, as well as in a real-time and long-term context.^8^,^9^

Early in this project, the application of informatics best practices was identified as a key requirement to ensure that the resulting technology solution would be effective. At its core, informatics takes into account human factors (processes and relationships) as well as technological factors to ensure successful outcomes.^10^–^12^ MNT4P worked in partnership with PHII to develop an informatics infrastructure as a national model for supporting the nutritional needs of patients living with IMDs. No human research was involved with the work described in this article.

## Approach

### Informatics methodology

When entering into any new informatics project for the purpose of improving health outcomes, PHII acts in accordance with its foundational Collaborative Requirements Development Methodology (CRDM). This methodology operates in 3 steps: (1) business process analysis—documenting how work is conducted in its current state; (2) business process redesign—highlighting inefficiencies of current processes, finding repeatable efficiencies, and restructuring workflows; and (3) requirements definitions—creating an exhaustive account of all functions and features a new system would need to contain (eg, functional requirements).^13^

## Planning and Deploying the Minimally Viable Product

The team planned to create a minimally viable product (MVP) (ie, the most basic working version) that not only would perform all the required functions upon launch but also could scale up in the future to perform additional services as they are identified and prioritized. To determine the needs for this scalable MVP, the project team conducted CRDM, as described earlier, in the summer of 2017 (see Supplemental Digital Content Table 2, available at http://links.lww.com/JPHMP/A731). Over the following year after completing CRDM, PHII conducted a vendor analysis to explore existing commercial off-the-shelf (COTS) data repository platform (DRP) solutions that could meet the collaboratively defined functional requirements (see Supplemental Digital Content Table 3, available at http://links.lww.com/JPHMP/A732).

By summer 2018, the team had used the vendor analysis to identify DrChrono as the most fitting COTS DRP solution available. The team further defined business processes based on DrChrono's capabilities and built a starting, functional version of the platform (ie, the MVP) that met those requirements. Training and practice sessions followed, and by February 2019, the system was live, including capabilities for tasks, charts, and clinical notes. A series of mini-pilots followed in the summer of 2019, and by January 2020, MNT4P staff had transitioned away from manual processes for enrolling patients and capturing and storing clinical data, making the MVP fully operational (see Supplemental Digital Content Figure 1, available at http://links.lww.com/JPHMP/A733).

## Results and Outcomes

The operational MVP includes patient profiles, scheduling, task assignment for staff, clinical notes and system attachments, custom exportable reports, and a patient portal for communication between MNT4P staff and patients (Table). Each digital health footprint contains patient demographic and diagnostic information, clinical notes, validated insurance information, and relevant flags added by staff indicating social determinants of health (eg, if a patient speaks English as a second language or has barriers to transportation).

**TABLE:** Impact of Data Collected Through the Digital Health Footprint

	Digital Footprint Component	Data Collected	Impact			
Interacting With Patients			Interacting With Providers	New Knowledge Generation	Quality Improvement
Now	Referral application	Referring provider information Reason(s) for referral Patient contact information		✓	✓	✓
	Enrollment form	Demographics Diagnostic information Nutrition needs Service needs (eg, patient education, insurance navigation)	✓	✓		✓
	Patient chart	Communication and interactions with program staff Clinical documents Insurance documents MNT4P notes Potential for exchange with interoperable EHRs	✓	✓	✓	✓
Future	Diet tracker	Historical diet records Compliance with medical food diet Abnormalities in diet Obstacles to maintaining diet compliance	✓	✓	✓	✓
	Outcomes tracker	Social outcomes Educational outcomes Clinical outcomes			✓	✓
	Insurance navigation tracker	Insurance denials and approvals categorized by diagnosis, insurance provider, etc	✓		✓	✓

Abbreviations: EHR, electronic health record; MNT4P, Medical Nutrition Therapy for Prevention.

The MVP currently contains digital health footprints of 373 patients living with IMDs in Georgia (see Supplemental Digital Content Figures 2, 3, and 4, available at http://links.lww.com/JPHMP/A734, http://links.lww.com/JPHMP/A735, and http://links.lww.com/JPHMP/A736, respectively). The system offers a HIPAA-compliant, efficient record-keeping system that has the potential to share data with other DRPs. The MVP also enables data standardization, improving ease of analysis and record harmonization, as well as ensuring cleaner data and reducing duplication as compared with the former manual processes. The MNT4P staff are able to rapidly access data as well as document and potentially exchange health information through interoperable electronic health records (EHRs), not only to improve operational efficiency but also to potentially contribute to new knowledge generation. The MVP is not currently exchanging data with other EHRs but has built-in Fast Healthcare Interoperability Resources (FHIR) and Health Level 7 (HL7) standards, which enable possible future electronic data exchange. The timing of the MVP's launch also expedited MNT4P's transition to a telehealth model in March 2020 in response to the global COVID-19 pandemic by making digitized, centralized patient records ready and available for uninterrupted patient care during the statewide lockdown.

## Discussion

The MVP provides a basic, standardized, and scalable infrastructure for the digital health footprint, improving on the almost entirely manual process MNT4P previously relied on, which combined Excel files, paper records, and the institutional memory of longtime staff. When newborns who receive a positive NBS are referred to MNT4P, a record is established that provides the capability for lifelong follow-up. Ongoing interventions and access to medical resources may prevent hospitalizations and adverse health outcomes stemming from noncompliance or loss to follow-up and, by extension, promote improved quality of health and decrease health care cost.

IMDs have the potential to affect all areas of a patient's life, and an IMD diagnosis means lifelong dietary primary treatment.^14^ Thus, any barrier to access of medical foods can result in patients simply going without and suffering the medical consequences. One of MNT4P's goals is to identify barriers to access and collaborate with key stakeholders to overcome those barriers. The digital health footprint will assist in flagging these challenges on an individual patient level so that support and interventions can be offered swiftly to prevent adverse medical events and prevent patient loss to follow-up, as well as informing future prevention efforts.

The MVP will also help with continuous service provision and quality improvement as MNT4P grows by offering scalability and increased efficiency. This program could serve as a national or even international model for IMD NBS, follow-up, and continuity of care. The digital health footprint also can serve as a reference to clinicians on individual patient health and behavior to assist in shaping individualized treatment strategies and allowing for systematic patient monitoring. There are minimal barriers inherent to deploying a system such as this one. However, any robust and successful implementation depends on a thorough understanding of the underlying processes the system must support, which would involve comprehensive staff training.

Currently, dependable clinical data on patients living with IMDs are sparse. Clinical studies are few, and evidence-based interventions are difficult to design. Historically, data around utilization of resources for the IMD community have been untracked nationally. This initiative represents one of the first to track IMD treatment cost and utilization data, as well as prescription use by patients, and how well treatment trends adhere to current guidelines by age. The novel data sets generated by the digital health footprint will generate new knowledge that will contribute to both the Georgia IMD community and the national IMD population, which, in turn, can inform future interventions and policies.

Digital health footprinting will not only support researchers and clinicians but also empower patients to take control of their own health and enable patient advocacy within the IMD community.

**Implications for Policy & Practice**

- *Community health outcomes*: Access to medical foods and services is essential to the well-being of those who live with IMDs, and by preventing gaps in access, MNT4P's digital health footprint can contribute to improved community health outcomes and prevent unnecessary medical costs.
- *Knowledge generation*: This system lays the foundation for evidence-based policies to help the IMD community by generating data that have never been accessible before. For example, the Medical Nutrition Equity Act, H.R. 251, would provide additional insurance coverage for patients with IMDs and is currently under consideration by Congress, but advocates are finding it difficult to enact without sufficient data backing up the need for this additional financial support for medical foods.
- *Replication*: The digital health footprint is a model that can be applied to many other states or to other rare chronic diseases. As MNT4P continues to work toward implementing this model in Georgia, other states and jurisdictions may wish to explore tools and platforms that will enable similar efforts serving patients living with IMDs and other diseases requiring specialized care. This model will ideally aid prediction, enable prevention, and empower providers to personalize care.

## Conclusion

The digital health footprint creates a bridge to close identified gaps for the IMD community, inform long-term follow-up for NBS, and supply critical data on needs for the community, all leveraging the principles and best practices of informatics and utilizing data from point of care. Rather than a one-and-done medical test, under this model, NBS serves as merely the first milestone along a path to lifelong health for individuals born with IMDs. The digital health footprint serves as a model approach to overcome challenges of access to medical nutrition therapy and to provide critical data for patients with IMDs or other rare genetic diseases.

## Supplementary Material

SUPPLEMENTARY MATERIAL
