# Supplementary material for: Using Informatics to Build a Digital Health Footprint of Patients Living With Inherited Metabolic Disorders Identified by Newborn Screening
Source: J Public Health Manag Pract. 2020 Nov 16;28(2):E340–4. doi: 10.1097/PHH.0000000000001250 (PMC8781221; doi:10.1097/PHH.0000000000001250)
Supplement: SUPPLEMENTARY MATERIAL [file jpump-28-e340-s001.docx]

*Table 2: Tools used by the project team to determine the MNT4P MVP*

|  |  | **Domain** | | |
| --- | --- | --- | --- | --- |
| **Tool** | **Description** | **People** | **Process** | **Tech** |
| Stakeholder analysis | One-on-one interviews with seven public health stakeholders, including leadership and frontline staff, captured the views of both big-picture strategists and direct practitioners; used to inform the business process analysis. | ✓ | ✓ |  |
| User stories | 11 user stories (i.e., a set of hypothetical future system users) included a deep dive of these fictional personas’ likes, pain points, and goals in terms of informatics. This human-focused assessment of future state complements the functional requirements definition. | ✓ | ✓ | ✓ |
| Business process analysis and redesign | In accordance with CRDM, an analysis of current business processes included documenting existing tasks and workflows, and identifying and analyzing common task sets. | ✓ | ✓ | ✓ |
| Functional requirements | 259 new functional requirements for a future-state system were all driven by function rather than form—that is, they define *what* the system should do, but do not dictate or limit *how* the system should do it. The team defined requirements based on how a future state system would ease manual burden, improve program operations, and generate new knowledge on the patient population. |  | ✓ | ✓ |
| Vendor analysis | A vendor analysis provided an inventory of existing electronic health record solutions that may meet the collaboratively defined functional requirements. | ✓ | ✓ | ✓ |
| Provider landscape analysis | The analysis of the current IMD landscape was informed by four meetings with medical food providers. The analysis focused on existing assistance and insurance navigation programs. |  | ✓ | ✓ |
| Provider wizard and MNT4P website | These public-facing tools provide information on the MNT4P program and help guide patients with metabolic disorders to the appropriate support resources. The interactive wizard provides guidance on obtaining medical foods and navigating insurance to secure coverage, and it serves as a decision support tool. The website allows new patients to enroll directly, or their provider to enroll them. | ✓ |  | ✓ |
