# Supplementary material for: Using Informatics to Build a Digital Health Footprint of Patients Living With Inherited Metabolic Disorders Identified by Newborn Screening
Source: J Public Health Manag Pract. 2020 Nov 16;28(2):E340–4. doi: 10.1097/PHH.0000000000001250 (PMC8781221; doi:10.1097/PHH.0000000000001250)
Supplement: SUPPLEMENTARY MATERIAL [file jpump-28-e340-s002.docx]

*Table 3: High-level vendor analysis comparison of DrChrono and the anonymized second solution considered*

| **Component** | **Details** | **DrChrono** | **System X** |
| --- | --- | --- | --- |
| User interface | Well designed and easily operated by staff members with minimal background in utilizing EHRs | Exceptional | Satisfactory |
| Robust patient charts | The patient charts contain the core elements required to support MNT4P activities and patient management | Exceptional | Satisfactory |
| Apple™ Integrations | Apple mobility partner for seamless integration with Apple hardware and software | Exceptional | Not Available |
| Open Application Programming Interface (API) and Software Development Kit (SDK) | Free access to healthcare API and SDK to leverage data in the EHR platform | Exceptional | Not Available |
| Form builder | User friendly form builder that utilizes simple functionality (i.e., drag and drop) | Satisfactory | Satisfactory |
| Report builder | User friendly report builder that utilizes simple functionality (i.e., drag and drop) | Satisfactory | Satisfactory |
| Configurable system | MNT4P is not a traditional medical practice and has unique needs that must be met by a configuring a COTS | Exceptional | Unsatisfactory |
| Implementation approach | MNT4P requires significant technical and operational support to implement a COTS | Satisfactory | Unsatisfactory |
| Support & maintenance | Plan/structure for ongoing assistance from company post implementation | Satisfactory | Unsatisfactory |
|  |  |  |  |
