# Supplementary figures and images for: Using Informatics to Build a Digital Health Footprint of Patients Living With Inherited Metabolic Disorders Identified by Newborn Screening
Source: J Public Health Manag Pract. 2020 Nov 16;28(2):E340–4. doi: 10.1097/PHH.0000000000001250 (PMC8781221; doi:10.1097/PHH.0000000000001250)

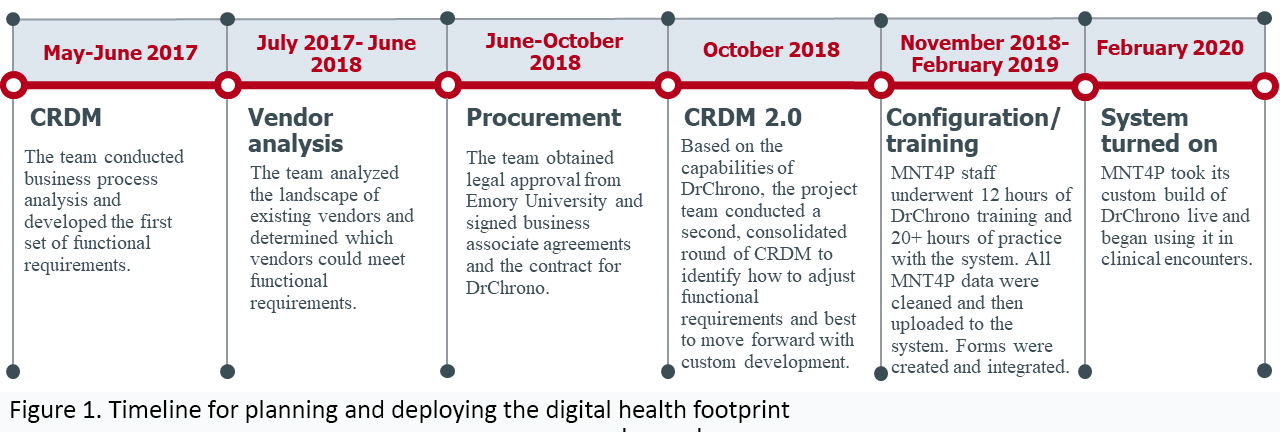

Supplement: SUPPLEMENTARY MATERIAL [file jpump-28-e340-s003.jpg]

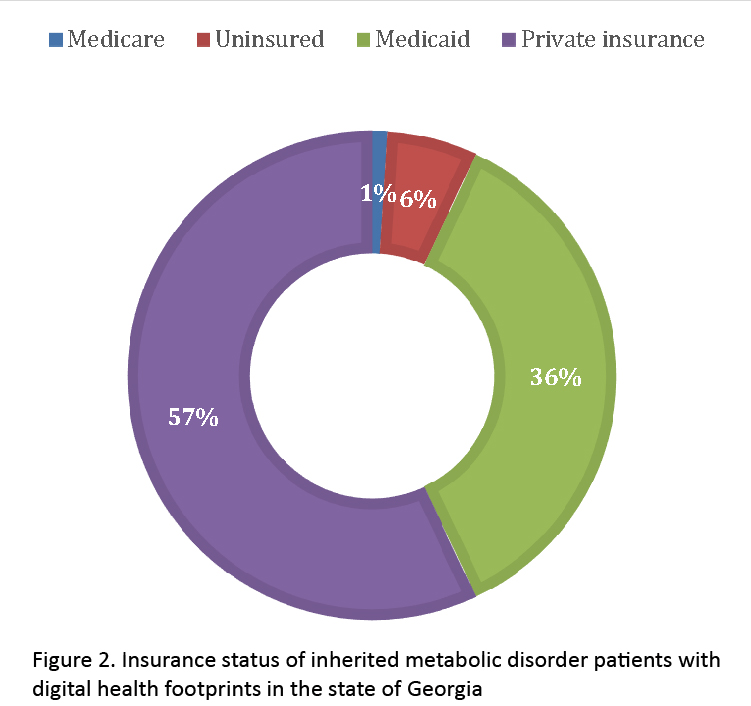

Supplement: SUPPLEMENTARY MATERIAL [file jpump-28-e340-s004.jpg]

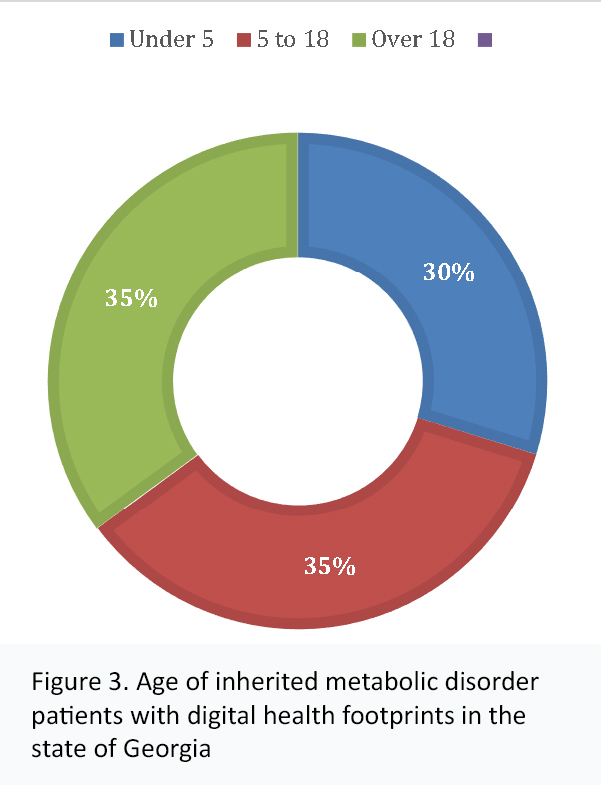

Supplement: SUPPLEMENTARY MATERIAL [file jpump-28-e340-s005.jpg]

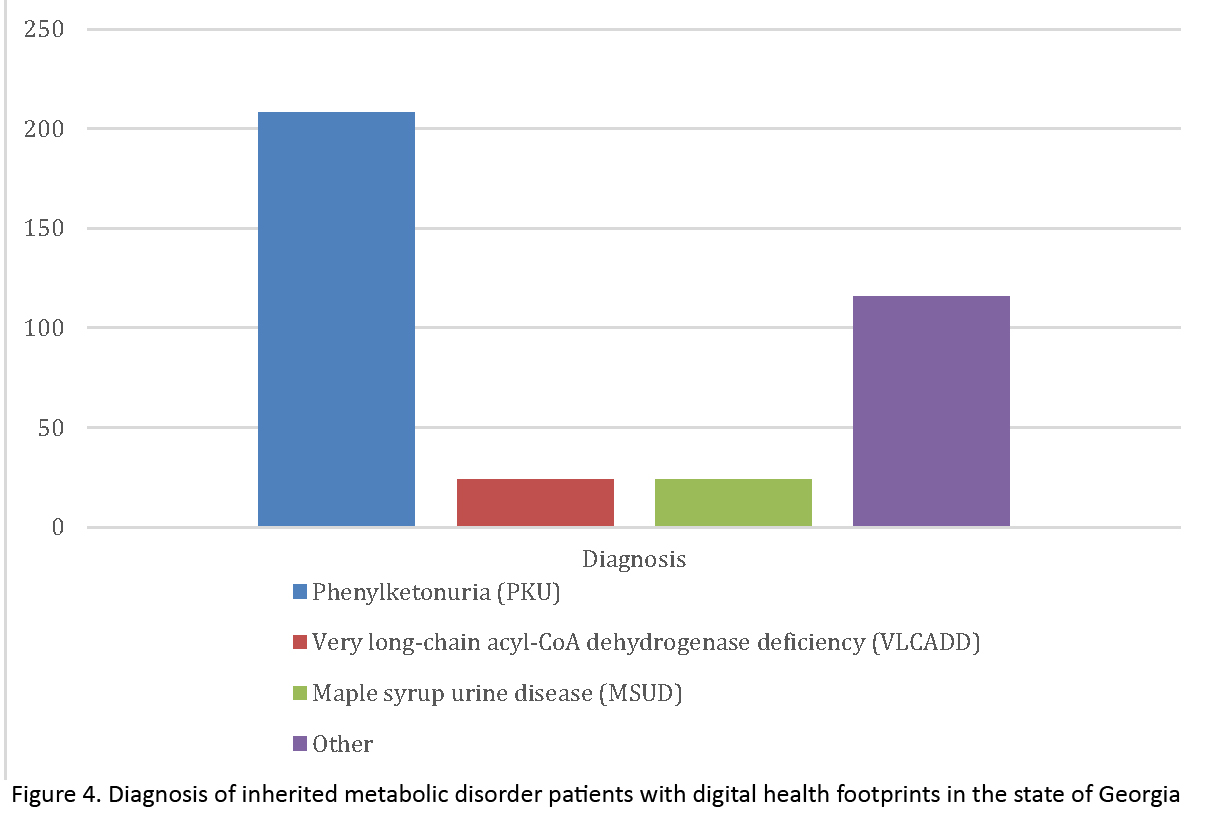

Supplement: SUPPLEMENTARY MATERIAL [file jpump-28-e340-s006.jpg]
